# Near Room‐Temperature Ferromagnetic Phase Achieved in 4*d* Ruthenate via Interfacial Ferrimagnetic Coupling and Octahedral Rotation Engineering

**DOI:** 10.1002/advs.202502613

**Published:** 2025-06-19

**Authors:** Mengqin Wang, Qinghua Zhang, Xiao Deng, Jie Zheng, Wenxiao Shi, Yunzhong Chen, Baogen Shen, Yuansha Chen, Tao Zhu, Fengxia Hu, Jirong Sun

**Affiliations:** ^1^ Beijing National Laboratory for Condensed Matter Physics and Institute of Physics Chinese Academy of Sciences Beijing 100190 China; ^2^ School of Physical Sciences University of Chinese Academy of Sciences Beijing 100049 China; ^3^ Spallation Neutron Source Science Center Dongguan 523803 China; ^4^ Ningbo Institute of Materials Technology & Engineering Chinese Academy of Sciences Ningbo Zhejiang 315201 China; ^5^ Songshan Lake Materials Laboratory Dongguan Guangdong 523808 China

**Keywords:** Ca_0.5_Sr_0.5_RuO_3_, interface engineering, LaMnO_3_, room‐temperature ferromagnetism, spin‐orbital coupling

## Abstract

Transition metal oxides (TMOs) simultaneously possessing strong spin‐orbit coupling, near room‐temperature ferromagnetism, and excellent conductivity are scarce while they show great potential applications in oxide spintronics. Here, a TMO with all these features is reported, existing as an interfacial phase in the 4*d* Ca_0.5_Sr_0.5_RuO_3_ layer sandwiched by two LaMnO_3_ layers. This phase is well conductive and ferromagnetic in a wide temperature range, with the highest Curie temperature of ≈275 K among the 4*d*/5*d*‐TMOs. Particularly, this interfacial phase shows a considerably improved saturation magnetization (≈2 *µ*_B_/Ru, twice that of the bulk counterpart), and a greatly reduced coercive force. All these features are highly desired by the application of spin‐orbit torque. Due to the presence of strong spin‐orbit coupling, such a Ca_0.5_Sr_0.5_RuO_3_ interfacial phase exhibits a significantly larger anomalous Hall conductivity than the typical 3*d* oxide ferromagnet La_2/3_Sr_1/3_MnO_3_ near room temperature. Analyses based on the techniques of X‐ray magnetic circular dichroism, polarized‐neutron reflectometry, and scanning transmission electron microscopy reveal a magnetic exchange interaction between the interfacial phases of Ca_0.5_Sr_0.5_RuO_3_ and LaMnO_3_ and an obvious expansion of the interfacial Mn─O─Mn bond angle, stabilizing the high‐temperature ferromagnetic state of the CSRO/LMO interface.

## Graphical abstract

Transition metal oxides combining strong spin‐orbit coupling, near‐room‐temperature ferromagnetism, and high conductivity are rare. A Ca_0.5_Sr_0.5_RuO_3_ interfacial phase between LaMnO_3_ layers has all these features, high *T*_c_, enhanced magnetization, and reduced coercivity.

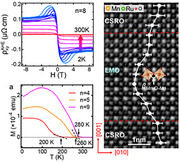

## 1 Introduction

Transition metal oxides (TMOs) with strong spin‐orbit coupling (SOC) have received wide attention due to their potential applications in the fields of oxide spintronics.^[^ ^1^, ^2^, ^3^, ^4^, ^5^, ^6^ ^]^ Among them, the TMOs with conductive and ferromagnetic (FM) properties are particularly attractive. For this kind of TMOs, as well established, the effect associated with spin‐orbit torque will be dramatically enhanced by anomalous Hall effect (AHE),^[^ ^7^, ^8^, ^9^ ^]^ resulting in an efficient spin injection and spin‐charge interconversion.

It is challenging to find materials that exhibit simultaneously a strong SOC, a high conductivity, and a good FM order up to room temperature while a *T*_C_ near or above room temperature is highly desired by practical applications. It is relatively easy to find a TMO with the latter two features. However, this kind of TMO is usually composed of 3*d* transition metals, showing a weak SOC (0.01–0.08 eV).^[^ ^10^, ^11^, ^12^ ^]^ It is therefore not a suitable spin current source. Noting the fact that the atomic SOC is proportional to Z^4^ (Z = atomic number of transition metals), TMOs composed of 4*d* or 5*d* transition metals have an obvious advantage over 3*d* TMOs as far as SOC is concerned. As reported, the energy scales of the atomic SOC are 0.1–0.3 eV and 0.3–0.8 eV for 4*d* and 5*d* transition metals, respectively.^[^ ^13^, ^14^ ^]^ Unfortunately, conductive 4*d*/5*d* TMOs are usually nonmagnetic, because of the formation of a broad *d*‐band, which depresses the on‐site Coulomb interaction and results in a weak electron correlation that cannot stabilize the FM state.^[^ ^15^, ^16^, ^17^ ^]^ To our knowledge, Ca_x_Sr_1‐x_RuO_3_ (CSRO) is the only exception in 4*d*/5*d* TMOs, showing metallic ferromagnetism. However, its Curie temperature is far below the desired room temperature (*T*_C_ ≤ 160 K).^[^ ^18^, ^19^ ^]^

In recent decades, intensive efforts have been devoted to inducing an FM order in originally nonmagnetic 4*d*/5*d* TMOs via interface engineering. The most representative works in this regard are those on 5*d* iridium oxides SrIrO_3_ and CaIrO_3_^[^ ^20^, ^21^, ^22^, ^23^, ^24^ ^]^ and 4*d* ruthenium oxide CaRuO_3._^[^ ^25^ ^]^ When grouping SrIrO_3_ (CaIrO_3_) with SrTiO_3_ (CaTiO_3_) to form superlattices (SLs), the interfacial layer of SrIrO_3_ (CaIrO_3_) was indeed driven into a magnetic state by the distinct tilt/rotation of the oxygen octahedra at the interface. However, the magnetic state of the SrIrO_3_ or CaIrO_3_ interfacial layer is antiferromagnetic, with a canted magnetic order as indicated by the low net magnetic moment (≈0.1 µ_B_/Ir), and the Curie temperature is low (<180 K). There were attempts to replace the nonmagnetic SrTiO_3_ (CaTiO_3_) layer with a magnetic layer, introducing interlayer exchange interaction. Although the interfaces become magnetic, no obvious improvements in magnetic order and Curie temperature were achieved in the resulting heterostructures. The typical examples are SrIrO_3_/LaMnO_3_ (LMO), SrIrO_3_/LaCoO_3_, SrIrO_3_/La_2/3_Sr_1/3_MnO_3_, SrIrO_3_/SrMnO_3_, and CaIrO_3_/CaMnO_3_.^[^ ^2^, ^26^, ^27^, ^28^, ^29^ ^]^ There were also attempts to combine CaRuO_3_ with SrTiO_3_ or CaMnO_3_, the results are not satisfactory either.^[^ ^2^, ^25^, ^26^, ^27^, ^28^, ^29^, ^30^, ^31^, ^32^ ^]^

Based on a survey of the literature, we found that, despite extensive investigations, the 4*d*/5*d* TMOs with good conductivity and FM ordering near room temperature have not been obtained. Here, we report on the discovery of an interfacial phase in the Ca_0.5_Sr_0.5_RuO_3_ (CSRO) layer sandwiched by two LMO layers. This phase is well conductive in a wide temperature range and ferromagnetic, exhibiting the highest Curie temperature (*T*_C_ ∼ 275 K) among the 4*d*/5*d* transition metal oxides. Particularly, this interfacial phase shows a saturation magnetization twice that of its bulk counterpart (≈2  *μ*_B_/Ru vs ≈0.7 μ_B_/R), a greatly reduced coercive force (≈0.05 T at 5 K), and a magnetic easy axis in the film plane. Due to the presence of strong SOC, the anomalous Hall conductivity $\sigma_{xy}^{AHE}\;$ of CSRO/LMO SLs is obviously larger than that of 3*d* oxide La_2/3_Sr_1/3_MnO_3_ films in the whole temperature range investigated. For example, as compared to the La_2/3_Sr_1/3_MnO_3_ films, the anomalous Hall conductivity of the CSRO/LMO SLs is increased by ≈100 times at 2 K, 26 times at 150 K, and five times at 280 K. Compared with the SrRuO_3_ bare films or the 5*d* oxide SL SrIrO_3_/LaCoO_3_, the $\sigma_{xy}^{AHE}$ of the interfacial CSRO phase displays an obvious advantage in the temperature range from 150 K to 280 K. Analyses based on the techniques of polarized neutron reflectometry (PNR), X‐ray magnetic circular dichroism (XMCD), and scanning transmission electron microscopy (STEM) indicate the occurrence of strong interlayer coupling which stabilizes the near room temperature FM state of CSRO.

## 2 Results and Discussion

### 2.1 Structural Analysis of CSRO/LMO Superlattice

**Figure 1:**
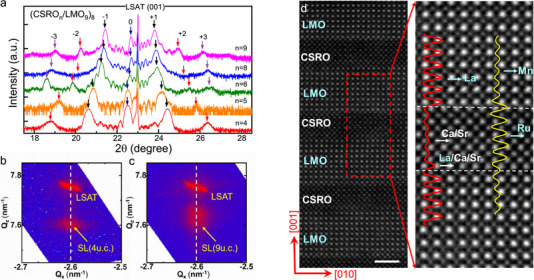
Structure characterization. a) X‐ray diffraction patterns of the CSRO_n_/LMO_9_ SLs on LSAT. Number 0 marks the (001) main peak of the SLs while else integers denote satellite peaks. b) and c) Typical reciprocal space mappings of the (103) reflection for the CSRO_n_/LMO_9_ SLs with *n* = 5 and 9, respectively. d) High‐angle annular dark‐field (HAADF) image of the cross‐section of the CSRO_5_/LMO_9_ SL, recorded along [100] zone axis. The LMO and CSRO layers are alternately stacked along the [001] direction, showing a high‐quality epitaxial growth. The scale bar in the bottom right corner represents 2 nm. The right panel is an enlarged image, with line profiles specifying the intensity of the A and B site atoms.

Five (CSRO_n_/LMO_9_)_8_ SLs were fabricated by alternately growing the LMO and CSRO layers on (001)‐oriented (LaAlO_3_)_0.3_(SrAl_0.5_Ta_0.5_O_3_)_0.7_ (LSAT) substrates with the technique of pulsed laser deposition. The layer thickness was fixed to 9 u.c. for LMO and *n* = 4, 5, 6, 8, or 9 u.c. for CSRO. The periodicity of the SL is 8. For simplicity, hereafter the SLs will be denoted as CSRO_n_/LMO_9_, meaning that each SL period has a *n*‐u.c.‐thick CSRO layer and a 9‐u.c.‐thick LMO layer. **Figure** **1**a shows the X‐ray diffraction (XRD) spectra of the SLs. In addition to the (001) main peak, distinct satellite peaks and thickness oscillation fringes are observed, indicating the high quality of the SLs. The (001) main peak is located at 22.5° when *n* = 4, corresponding to an out‐of‐plane lattice constant of 3.948 Å, and shifts to high angles with the increase of the layer thickness of CSRO. The latter feature is understandable considering the fact that the lattice parameter of CSRO is smaller than that of LMO (≈3.89 vs ≈3.91 Å).^[^ ^33^, ^34^ ^]^ From the separation between satellite peaks, the thickness of each period can be determined, and it is found to be consistent with the preset value.Figure 1b,c show the reciprocal space mappings (RSMs) of the (103) reflection, collected from the representative samples of CSRO_5_/LMO_9_ and CSRO_9_/LMO_9_, respectively. At first glance, the diffraction spots of the SLs are located just below that of the substrate, without in‐plane lattice relaxation, i.e., SLs suffer from a coherent strain with substrates. Figure 1d presents the representative high‐angle annular dark‐field (HAADF) image of CSRO_5_/LMO_9_, recorded with the technique of scanning transmission electron microscopy (STEM). As expected, the LMO and CSRO layers are alternately stacked along the [001] direction, showing a high‐quality epitaxial growth. As suggested by the line profile analysis along the A‐ and B‐site columns, respectively marked by yellow and red lines, the upper LMO/CSRO interface is atomically sharp, without visible interlayer mixing. At the bottom CSRO/LMO interface, in contrast, partial Ca^2+^ (Sr^2+^) ions in the first Ca/Sr–O monolayer of CSRO are replaced by La^3+^ ions as marked in Figure 1d, resulting in an A‐site intermixing within one unit cell of the CSRO layer. However, the peak intensities of La^3+^ and Mn^3+^ remain constant across the LMO layer, indicating the absence of ionic diffusion from the CSRO layer to the LMO layer. This result rules out the possible formation of a La_1‐x_(Sr, Ca)_x_MnO_3_ interfacial layer. To get further information on the elemental distribution across the interface, spatially resolved electron energy loss spectroscopy (EELS) element mapping was measured around the La‐*M*_4_,_5_, Ca‐*L*_2_,_3_, Mn─*L*_2_,_3_, Sr‐*L*_2_,_3_, Ru‐*M*_4_,_5_, and O‐*K* edges (see Figure S1, Supporting Information). The EELS mapping shows chemical uniformity within each layer of the SL. All these results specify the well‐ordered structure of the CSRO_n_/LMO_9_ SLs, with target layer thickness for each period.

### 2.2 Electronic Transport Properties

**Figure 2:**
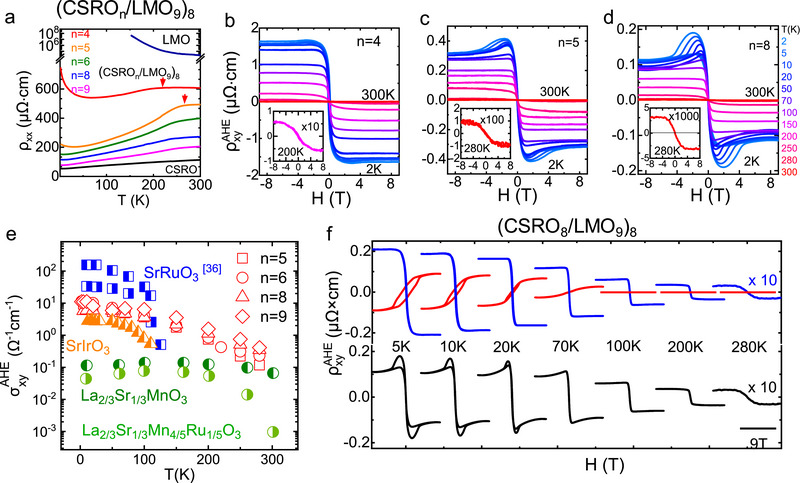
Transport properties of the CSRO_n_/LMO_9_ SLs. a) Longitudinal resistivity as a function of temperature (ρ_*xx*_−*T*). Results of the CSRO and LMO bare films were also presented for comparison. b)‐d) Anomalous Hall resistivity as a function of magnetic field ($\rho_{xy}^{AHE}$−*H*), obtained in the temperature range from 2 to 300 K for the samples of CSRO_n_/LMO_9_ SLs. From b) to c) and to d), the corresponding n is 4, 5 and 8. Step‐shaped $\rho_{xy}^{AHE}$‐*H* curves are observed when cycling the magnetic field between ± 9 T at low temperatures, indicating the establishment of FM order in the CSRO layer. The inset plot is a close view of the $\rho_{xy}^{AHE}$−*H* relation at an elevated temperature. Notably, the AHE of the interfacial remains sizable up to 280 K as indicated by the inset plots in c) and d). e) The saturation anomalous Hall conductivity ($\sigma_{xy}^{AHE}$) as a function of temperature for the SLs (n = 5, 6, 8, and 9), marked by red symbols. Data of representative magnetic oxides are also presented for comparison. The comparison data are taken from Hua et al.^[^ ^35^ ^]^ for La_0.67_Sr_0.33_MnO_3_ and La_0.67_Sr_0.33_Mn_0.8_Ru_0.2_O_3_, from Tian et al.^[^ ^36^ ^]^ for SrRuO_3_, from Arun et al.^[^ ^37^ ^]^ for SrIrO_3_/LaCoO_3_ SL. **f**) Upper panel: Two sets of AHE curves of the inner (red curve) and interfacial (blue curve) phases for the sample of CSRO_8_/LMO_9_. Bottom panel: Corresponding experiment results that can be well reproduced by a simple sum of the two AHE curves of the upper panel. Notably, the AHE of the interfacial phase remains sizable up to 280 K.

To explore the effect of interface engineering, we investigated the electronic transport properties of the CSRO_n_/LMO_9_ SLs. **Figure** **2**a shows the longitudinal resistivity ρ_*xx*_ as a function of temperature. Results of the CSRO and LMO bare films are also presented for comparison. The resistivity of the SLs exhibits a monotonic decrease as the layer thickness of CSRO grows, reducing by a factor of ≈3 from the samples of *n *= 4 to *n *= 9 (*T *= 300 K). All samples, except for CSRO_4_/LMO_9_, exhibit a metallic behavior in the temperature range from 2 to 300 K, similar to the CSRO bare film. For the CSRO_4_/LMO_9_ SL, a resistance upturn is observed at low temperatures, suggesting the occurrence of weak localization due to enhanced interfacial scattering when the CSRO layers are ultrathin. A remarkable observation is the inflection of the ρ_*xx*_ **‐*T*** curves, appearing at ≈200 K for the SL with *n *= 4 and ≈270 K for the SL with *n *= 5. This resistive anomaly reminds us of SrRuO_3_ which experiences a sizable resistivity drop when it transits from the paramagnetic to the FM state.^[^ ^38^ ^]^ It may be a signature of the FM transition of the CSRO layer. It could be an interfacial effect since the resistive inflection becomes ambiguous when *n* is thick.

Here we would like to emphasize that the transport properties of the SLs should be solely determined by the CSRO layers. As evidenced by the analysis of STEM, no La_1‐x_(Sr,Ca)_x_MnO_3_ interfacial layer is formed. This rules out intermixing‐induced Mn^4+^ ions. Moreover, the X‐ray absorption spectra (Figure S2, Supporting Information) indicate the absence of charge transfer at the CSRO/LMO interface. These results suggest that the Mn ions in the LMO sublayer are in the 3+ valence state, like the Mn ions in the LMO bare film, and the double exchange between Mn^3+^ and Mn^4+^ is not supported in either the inner or the interfacial layer of LMO. As a consequence, the LMO layer is insulated.

AHE is a fingerprint of the FM order, appearing in the materials with broken time‐reversal symmetry and SOC. In general, the Hall resistivity of a ferromagnet can be described by ρ_*xy*_ = *R_o_* · *H* + μ_0_ *R_s_*  · *M*. The first term is the ordinary Hall effect, which varies linearly with magnetic field (*H*), and the second term is AHE, which is proportional to spontaneous magnetization (*M*).Figure 2b–d shows the anomalous Hall resistivity $\rho_{xy}^{AHE}$ as a function of *H* for the CSRO_n_/LMO_9_ SLs with respectively *n *= 4, 5, and 9, obtained at different temperatures. Step‐shaped $\rho_{xy}^{AHE}$‐*H* curves are observed when cycling magnetic field between ± 9 T, indicating the occurrence of AHE thus the establishment of the FM order in the CSRO layer. Notably, the AHE solely comes from the CSRO layer since the LMO layer is insulated as aforementioned. The AHE is strong at low temperatures and weakens with the increase in temperature. However, it remains sizable up to the temperature of ≈200 K, for the sample of CSRO_4_/LMO_9_, and ≈280 K, for the samples with thick CSRO layers (see the inset plots inFigure 2b–d), close to the Curie temperature (*T*_C_) suggested by the ρ_*xx*_ **‐*T*** curve. As a summary, in Figure S3 (Supporting Information) we present the saturation value of $\rho_{xy}^{AHE}$ as a function of temperature. Defining the Curie temperature (*T*_C_) as the temperature where $\rho_{xy}^{AHE}$ vanishes, it will be ≈220, ≈280, and ≈300 K for the CSRO layers with the thickness of 4, 5, and 6 u.c. *T*_C_ remains ≈300 K when *n* exceeds six. As will be shown later, the Hall effect overestimates *T*_C_, and the upper bound of *T*_C_ is ≈275 K, deduced from the data of direct magnetic measurements. Notably, 275 K is the highest Curie temperature obtained so far for the 4*d*/5*d* TMOs which host a strong SOC, more than ≈100 K higher than previously reported values.^[^ ^26^, ^27^, ^37^ ^]^ It is interesting that the Curie temperature of the CSRO layer is much higher than that of either the CSRO or LMO bare film (≈70 or 160 K, see Figure S4, Supporting Information). To further reveal the distinct features of the interfacial CSRO layer with the FM order, inFigure 2e we compare the saturation anomalous Hall conductivity $\sigma_{xy}^{AHE}$=$\rho_{xy}^{AHE}\;/\rho_{xx}^{2}$ of the CSRO/LMO SLs with typical FM oxide films. Due to the presence of strong SOC, the $\sigma_{xy}^{AHE}$ of CSRO/LMO SLs is obviously larger than that of the 3*d* oxide La_2/3_Sr_1/3_MnO_3_ in the whole temperature range investigated. The largest enhancement rate of $\sigma_{xy}^{AHE}$ of the CSRO/LMO SLs, compared to the La_2/3_Sr_1/3_MnO_3_ bare film, is ≈100 at 2 K, ≈26 at 150 K and ≈5 at 280 K. Previous work reported that Ru doping in La_2/3_Sr_1/3_MnO_3_ film could enhance the anomalous Hall resistivity $\rho_{xy}^{AHE}$. However, the random Ru doping also severely deteriorates the longitudinal conductivity ρ_*xx*_ of the film, resulting in even lower $\sigma_{xy}^{AHE}$ than that of the undoped films. As for the comparison with SrRuO_3_ bare films or SrIrO_3_/LaCoO_3_ SL, the $\sigma_{xy}^{AHE}$ of the interfacial CSRO phase in the CSRO/LMO SLs displays an obvious advantage in the temperature range from 150 to 300 K, due to its dramatically improved *T*_C_. One thing deserving special attention is the appearance of hump‐shaped $\rho_{xy}^{AHE}-H$ curves in the samples with thick CSRO layers (Figure 2c,d). This anomaly emerges when the samples are cooled below ≈70 K and develops with further cooling. It resembles the AHE of two coexisted FM phases with opposite anomalous Hall coefficients. After a direct calculation, the complex AHE is indeed decomposed into two $\rho_{xy}^{AHE}-H$ curves with opposite *R*_S_. As an example, inFigure 2f we show the two deduced AHE curves for the typical sample CSRO_8_/LMO_9_ (upper panel). The sum of these two curves well reproduces the experimental results (bottom panel). According to Figure 2f, the first FM phase (red curves) exhibits a positive Hall coefficient *R*_S_ and a Curie temperature of ≈70 K whereas the second FM phase (blue curves) shows a negative *R*_S_ and a Curie temperature close to 300 K. A further difference is the coercive field, which is ≈1.2 T ( = 5 K) for the first FM phase and ≈0.05 T (*T *= 5 K) for the second FM phase. (Figure S5, Supporting Information) Considering the fact that the AHE of the SL comes exclusively from the CSRO layer, the two FM phases certainly bear a close relation to CSRO. We further investigated the Hall effect of a CSRO bare film (≈30 u.c. in thickness) and found evidence that the first phase is the CSRO phase distant from the interface (Figure S6, Supporting Information). This explains the enhancement of the AHE hump as the layer thickness of CSRO increases (Figure 2c,d). Of special interest is the appearance of the second FM phase, it could be an interfacial phase: The CSRO/LMO interlayer coupling has resulted in an emergent FM phase with a much higher *T*_C_, a greatly reduced coercive force, and a strong but negative *R*_S_, totally different from the CSRO bulk phase. Considering the fact that the hump‐shaped feature is visible in the sample of CSRO_5_/LMO_9_ but invisible in the sample of CSRO_4_/LMO_9_, we can safely say that the effective thickness of the interfacial layer is ≈2 u.c.

In general, hump‐shaped $\rho_{xy}^{AHE}-H$ curves could be produced by topological spin textures. To check whether topological spin textures exist or not, we conducted the magnetic force microscope (MFM) measurements for the sample of CSRO_9_/LMO_9_ SL. The MFM images were obtained at 2 K with the applied magnetic fields of 1 T, 2 T, and 3 T, respectively. Under these fields, the Hall hump is most pronounced. As shown in Figure S7 (Supporting Information), no topological magnetic structures similar to skyrmions were observed. Therefore, the hump‐shaped Hall anomaly in the $\rho_{xy}^{AHE}-H$ curves cannot be ascribed to topological Hall effect.

### 2.3 Magnetic Anisotropy

**Figure 3:**
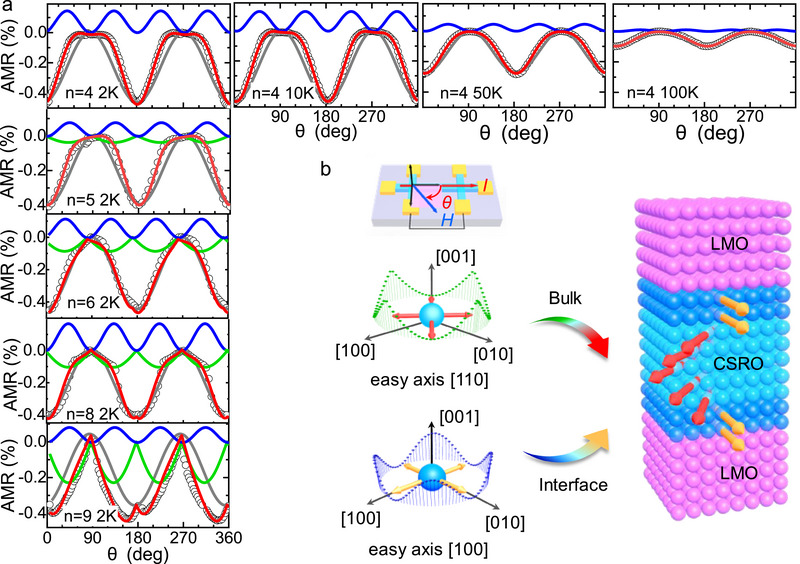
Anisotropic magnetoresistance. a) Anisotropic magnetoresistance obtained by rotating magnetic field in the (001) plane while keeping the magnitude of the magnetic field at 3 T. Data in the top panel are recorded at selected temperatures of 2, 10, 50, and 100 K for the sample of CSRO_4_/LMO_9_. The left column is the anisotropic magnetoresistance of different samples, collected at a fixed temperature of 2 K. Black circles represent experimental data. Red lines are the results of curve fitting. Colorful dashed lines are different components of the AMR oscillation. Inset is the geometry for AMR measurements. The electric current is applied along the [100] direction and the magnetic field rotates in the (001) plane, forming an angle of θ with the applied current. b) From top to bottom: a schematic diagram of the experimental setup, a 3D trajectory of the AMR of the inner and the interfacial phases. Arrows in the figure mark the directions of magnetic easy axes. The right panel of b) is a schematic diagram showing the CSRO layer sandwiched by two LMO layers and the evolution of the magnetic easy axis.

The above investigations indicate the formation of an interfacial phase in the CSRO layer. This phase is conductive and FM up to room temperature and, more than that, may have a moderate SOC due to the large atomic SOC of Ru atoms.^[^ ^13^ ^]^ In fact, ruthenium oxides such as SrRuO_3_, CaRuO_3_, and RuO_2_ usually show a strong SOC, as suggested by the strong spin Hall effect.^[^ ^39^, ^40^, ^41^, ^42^, ^43^ ^]^ To get further knowledge about this phase, we measured anisotropic magnetoresistance (AMR) which will give us information on magnetic anisotropy. According to the results of magnetic measurements, the magnetic easy axis of the SLs is in the *x‐y* plane (Figure S8, Supporting Information). Therefore, only the AMR resulting from a rotating magnetic field in the *x‐y* plane was presented. The AMR is defined by AMR = (*R*_θ_−*R*_90°_)/*R*_90°_, where *R*_θ_ is the resistance measured with an in‐plane field (3 T) that forms an angle of *θ* with the [100] direction of the applied current. The angle‐dependent AMR is shown in **Figure** **3**a for the sample of CSRO_4_/LMO_9_ which has only an interfacial CSRO phase, measured at selected temperatures between 2 and 100 K. The AMR‐*θ* curves are somewhat complex. The AMR is negative when *θ* = 0, exhibiting a value of −0.5%. It grows rapidly with the increase of *θ*, achieving a plateau around zero when *θ* takes the angles from ≈60° to ≈90°. Further increasing *θ* leads to rapid decrease in AMR until the appearance of a deep valley around *θ*  = 180°. Sweeping *θ* from 180° to 360°, AMR repeats the behaviors from 0° to 180°.

Fortunately, a careful analysis indicates that the experiment results can be well reproduced by a theoretical simulation based on the formula AMR  = *a*_2_  × cos(2*θ* − ω_2_) + *a*_4_ × cos(4*θ* − ω_4_), adopting suitable fitting parameters. Here *a*_2_ and *a*_4_ are the amplitudes of twofold and fourfold oscillations, respectively, and ω_2_ and ω_4_ are the corresponding offset angles. Two types of oscillations have been reported in the literature, where the twofold oscillation was ascribed to normal AMR or the effect of Lorentz scattering, and the fourfold oscillation to magnetocrystalline anisotropy.^[^ ^44^, ^45^ ^]^ Based on the analysis of the fourfold oscillation, we concluded that the [100], [‐100], [010], and [0‐10] axes are the magnetic easy axes of the interfacial CSRO phase: along these directions, AMR valley appears due to the suppression of spin‐flip scattering.

The left column ofFigure 3a presents the *AMR*‐*θ* curves of different SLs at a constant temperature of 2 K. It is found that the CSRO_4_/LMO_9_ is the only sample whose AMR can be fitted by two AMR components, i.e., twofold and fourfold oscillations. When the layer thickness of CSRO exceeds 4 u.c., an additional term of |*a*_3_ × cos(2θ − ω_3_)| is required to describe the *AMR*‐*θ* relation. At first glance, the contribution from this additional term emerges when *n *= 5 and develops with the increase of *n*. Further analysis shows that this AMR term results in a broad valley in the [110], [‐110], [‐1‐10], or ^[^ ^1^, ^2^, ^3^, ^4^, ^5^, ^6^, ^7^, ^8^, ^9^, ^10^ ^]^ direction and a sharp tip along the [010], [‐100], [0‐10], or [100] direction. These are exactly the AMR behavior of the CSRO bare films (Figure S9, Supporting Information). Therefore, a bulk‐like CSRO phase appears when the CSRO layer is thicker than 4 u.c., supporting the conclusion of the AHE investigations. A further remarkable observation is the distinct magnetic easy axis of this bulk‐like phase, which aligns along the [110], [‐110], [‐1‐10], or ^[^ ^1^, ^2^, ^3^, ^4^, ^5^, ^6^, ^7^, ^8^, ^9^, ^10^ ^]^ direction, different from the interfacial phase. In general, the different magnetic anisotropy of the interfacial and inner CSRO phases will result in a non‐collinear spin texture (Figure 3b). Therefore, it is a feasible approach to modulate spin texture by adopting the appropriate layer thickness of CSRO for the CSRO_n_/LMO_9_ SLs.

### 2.4 Magnetization Depth Profile of the (CSRO_9_/LMO_9_)_5_ SL

**Figure 4:**
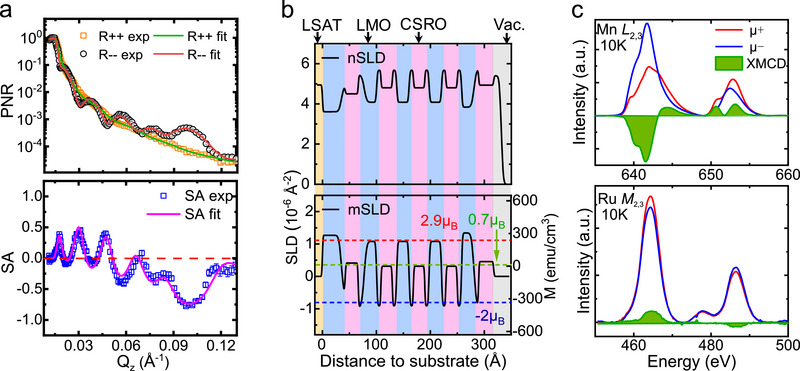
Magnetization depth profiles of the (CSRO_9_/LMO_9_)_5_ SL. a) Reflectivity curves for spin‐up (R^++^) and spin‐down (R^–^) polarized neutrons (upper panel), normalized to the asymptotic value of the Fresnel reflectivity (R_F _= 16π^2^/*q* ^4^). The measurements were performed at 10 K in an applied in‐plane field of 1.2 T. The large splitting between R^++^ and R^−^ suggests a large net magnetic moment across the entire sample. The spin asymmetry (SA) was described by (R^++^−R^–^)/(R^++^+R^–^) (bottom panel). Open symbols and solid lines are experimental data and the results of best curve fitting, respectively. Error bars represent the standard deviation. **b)** Depth profiles of nuclear scattering length density (nSLD) and the magnetic scattering length density (mSLD), adopted to get the best curve fitting presented in a). c) Mn *L*_2,3_ and Ru *M*_2,3_ edge X‐ray absorption spectra of the CSRO_3_/LMO_3_ sample, measured with a left‐handed (red curve) or a right‐handed (blue curve) circularly polarized light. The green lines and shaded areas show the XMCD spectra and the inset shows antiferromagnetic coupling between Mn and Ru at the interface. The sample was first cooled to a preset temperature 10 K in a magnetic field of 5 T and then measured after the thermal stabilization state was reached.

To clarify the magnetic profile across the CSRO/LMO interfaces, we performed the experiments of PNR for the sample of (CSRO_9_/LMO_9_)_5_. As a function of wave vector transfer *q *= 4𝜋sin𝜃/𝜆, **Figure** **4**a (upper panel) shows the neutron reflectivity, obtained at 10 K with the neutron spin parallel (R^++^) or antiparallel (R^−^) to the in‐plane magnetic field (1.2 T), where 𝜃 is the incident angle of the neutron beam and 𝜆 is neutron wavelength. A clear first‐order Bragg reflection appears at *q≈*0.1 Å^−1^, manifesting the uniformity of the sample with sharp interfaces. The bottom panel of Figure 4a shows the spin asymmetry defined by SA = (R^++^−R^−^)/(R^++^+R^−^), which carries the information about the depth variation of the net magnetization across the SL. The best fittings of the PNR and SA data are represented by the solid lines inFigure 4a, based on the model inFigure 4b, which illustrates the nuclear scattering length density (nSLD) and magnetic scattering length density (mSLD). The perfect consistency between experimental data and curve fitting confirms the reliability of the nuclear and magnetic models. Remarkably, the mSLD profile shows that each CSRO layer is divided into three sublayers, with different magnetic moments and directions. The two interfacial layers exhibit a high magnetization (≈2 *µ*_B_/Ru) whereas the inner layer shows a low magnetization close to that of the CSRO bare film (≈0.7 *µ*_B_/Ru). The average thickness of the interfacial layers is found to be ≈2 u.c., well consistent with the value suggested by the investigations of the AHE. A further remarkable observation is the antiparallel alignment of the magnetic moments of the CSRO interfacial layer and the LMO layer. This result reminds us of the antiferromagnetic interlayer exchange between SrRuO_3_ (SRO) and La_0.7_Sr_0.3_MnO_3_ (LSMO).^[^ ^46^ ^]^ It could be a general feature of the magnetic coupling between ruthenates and manganites.^[^ ^30^ ^]^ The magnetization is ≈2.9 *µ*_B_/Mn for the middle LMO layers in the SL. Considerable magnetic enhancement is observed in the first and last LMO layers, which can be ascribed to the effects of boundary conditions: The first layer has an interface with the substrate and the last layer has only a CSRO cap layer. To further confirm the antiferromagnetic coupling between the CSRO interfacial layer and the LMO layer at the interface, we performed XMCD measurements around the Mn *L*_2,3_ edge and Ru *M*_2,3_ edge of the CSRO_3_/LMO_3_ SL.Figure 4c shows the X‐ray absorption spectra measured with a left‐handed (*µ* ^−^) and a right‐handed (*µ* ^+^) circularly polarized light, respectively. The XMCD spectra were obtained by calculating the differences between the *µ* ^+^ and *µ* ^−^. As expected, the XMCD signal of the Ru *M*_2,3_ edge is opposite to that of the Mn *L*_2,3_ edge, consistent with the antiparallel spin alignment between interfacial Ru and Mn ions as revealed by the PNR analysis. This is similar to previous studies on the ruthenate/manganite heterojunctions,^[^ ^30^, ^46^, ^47^ ^]^ where the antiferromagnetic superexchange interaction between Ru^4+^ and Mn^3+^ ions was considered as the reason. Ziese et al. reported that, for the LSMO/SRO superlattices, when keeping the LSMO layer thickness at a constant of 1.6 nm (≈4 u.c.), the coercive field of the SRO layer would increase with reduced thickness.^[^ ^46^, ^47^ ^]^ For example, the coercive field is ≈2 T for the 5‐nm‐thick (≈12.5 u.c.) SRO sublayers and ≈4 T for the 3 nm‐thick (≈7.5 u.c.) SRO layer. For our CSRO_3_/LMO_3_ SL, it is possible that the 3‐u.c.‐thick CSRO sublayer is strongly pinned by interfacial coupling and does not rotate towards field direction even in the field as high as 5 T. All these results suggest that the CSRO interfacial layer and the LMO layer are magnetically antiparallel, forming a ferrimagnetic magnetic structure.

### 2.5 Macroscopic Magnetic Properties

**Figure 5:**
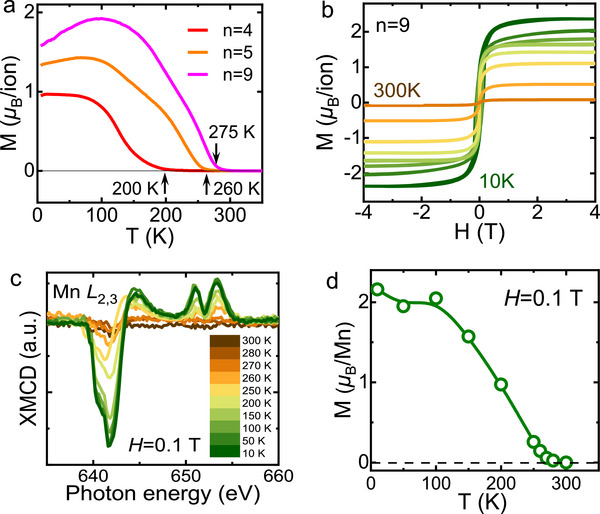
Macroscopic magnetic properties. a) Temperature dependence of the magnetization of (CSRO_n_/LMO_9_)_8_, measured with an in‐plane field of 0.05 T in field‐cooling mode. The downward magnetic turn at low temperatures comes from the CSRO inner phase whose spin is antiparallel to that of the LMO layer. b) Magnetic hysteresis loops of the CSRO_9_/LMO_9_ SL, measured in the temperature range from 10 to 300 K. c) XMCD spectra of the Mn *L*_2,3_ edge, collected from 10 to 300 K. The sample was first cooled to 10 K in a magnetic field of 0.8 T, and then measured after reducing the applied filed to 0.1 T at different temperatures. d) Magnetization as a function of temperature, deduced from the XMCD spectra of the LMO layer. Due to the overlap of the *L*_2_ and *L*_3_ edges of Mn, there is an error in the spin sum rule. When calculating the spin moment, the number of holes we used is six, and the correction factor is 0.64, adopted from previous works.^[^ ^48^, ^49^, ^50^ ^]^

The above investigations confirm the formation of a distinct interfacial phase in CSRO. According to the results of AHE, this layer is FM up to room temperature. This is amazing since neither the CSRO (*T*_C_ ∼ 70 K) nor the LMO (*T*_C_ ∼ 160 K) bare film exhibits such a high *T*_C_. To get an insight into the mechanism of this magnetic anomaly, knowledge about the LMO layer is required. Noting that the information on LMO cannot be obtained from transport experiments since the LMO layer is insulated, we performed direct magnetic measurements. In **Figure** **5**a we show the magnetization as a function of temperature (*M‐T*) for the CSRO_n_/LMO_9_ superlattices with an in‐plane magnetic field of 0.05 T (*n *= 4, 5, and 9 u.c.). The *M*‐*T* curves show the typical thermomagnetic behavior of the magnetic materials, indicating the establishment of the FM order below a critical temperature. The Curie temperature can be deduced from these *M‐T* curves using the two‐tangent method, and it is ≈200 K for *n *= 4, ≈260 K for *n* = 5, and ≈275 K for *n *= 9, which is a little bit lower than that deduced from the AHE. To understand this difference, we further measured the magnetic hysteresis loops at different temperatures for the typical sample CSRO_9_/LMO_9_ (Figure 5b), with in‐plane magnetic fields (The magnetic easy axis is in the film plane as shown in Figure S8, Supporting Information). The corresponding results for other samples are presented in Figure S10 (Supporting Information). As expected, magnetic hysteresis loops are clearly seen at low temperatures, indicating the emergence of the FM order in the SL samples. With the increase of temperature, however, the magnetic hysteresis loop shrinks and collapses into a step shaped *M*‐*H* relation when temperature exceeds 250 K. A further analysis indicates that the *M*‐*H* curves at 280 K and 300 K can be well described by the Langevin function, implying the appearance of superparamagnetic state at high temperatures^[^ ^51^, ^52^ ^]^ (Figure S11, Supporting Information). It is therefore clear that the SL sample indeed owns a Curie temperature much higher than that of the LMO bare film, as suggested by the AHE. For example, *T*_C_ is ≈275 K for the CSRO_9_/LMO_9_ SL whereas it is only ≈160 K for the LMO plain film. As for the AHE above 275 K, it may be a super‐paramagnetic behavior, i.e., the CSRO layer in CSRO_9_/LMO_9_ is superparamagnetic above 275 K.

In general, the magnetic behaviors of the SL are jointly determined by the CSRO and LMO layers. However, the contribution of the LMO phase cannot be determined simply based on the results of macroscopic measurements, and we even have no idea about whether or not the LMO layer will maintain the FM order up to 275 K, like the CSRO interfacial phase. To get the information specific to LMO, we performed a further analysis of the XMCD spectra around the Mn *L*_2_,_3_ edges of CSRO_3_/LMO_3_. Figure S12 (Supporting Information) presents the X‐ray absorption spectra measured with a left‐handed (*µ* ^−^) and a right‐handed (*µ* ^+^) circularly polarized light from 10 K to 300 K. Here only the data of the XMCD spectra are shown (Figure 5c). As expected, a strong dichroism is observed when the temperature is low, indicating the establishment of a highly ordered magnetic state in the LMO layer. Fascinatingly, the XMCD signal remains strong at the Curie temperature of the LMO bare film (≈160 K), and visible even at 270–280 K. These are signatures of the stabilization of the FM state in the LMO layer. To get a quantitative idea about the evolution of the FM order, inFigure 5d we show the deduced magnetization as a function of temperature. The magnetization takes a value of ≈2.2 *µ*_B_/Mn at 10 K, and undergoes a smooth decrease from ≈2.2 *µ*_B_/Mn to ≈2.0 *µ*_B_/Mn when temperature grows from 10 to 100 K. Further increase in temperature leads to first a rapid and then a slow decrease in magnetization. XMCD signals vanish above 280 K. Notably, this magnetization‐temperature relation reproduces the main features of the *M*_S_‐*T* curves of CSRO_3_/LMO_3_ (Figure S13, Supporting Information), manifesting the important contribution of the LMO layer to the magnetic moment. The most remarkable observation here is that interface engineering affects not only CSRO but also LMO, enhancing the FM order in both layers. It is a plausible assumption that the interlayer coupling improves the FM order of LMO, and the improved LMO layer magnetizes the CSRO layers in return.

Notably, the maximal magnetization deduced from the XMCD data is ≈2.2 *µ*_B_/Mn, obviously lower than that suggested by PNR (∼2.9 *µ*_B_/Mn). It is possible that the LMO layer is not in the magnetic saturation state under an applied field of 0.1 T.

### 2.6 Discussion

The X‐ray absorption spectra indicate that the Mn ions of the SL show exactly the same valence state as that of the LMO bare film, without interlayer charge transfer (Figure S2, Supporting Information). Therefore, the improved *T*_C_ of the LMO layer in the SL cannot be ascribed to the change in the valence state of Mn ions. In the perovskite manganite oxides, the *d* electrons of the Mn ion form a covalent bond with the *p* electrons of the intermediate O anion. It has been previously reported that the Mn─O bond length and Mn─O─Mn bond angle have a strong influence on the strength of the exchange interactions, modulating the magnetic properties of manganite oxides.^[^ ^53^, ^54^, ^55^, ^56^, ^57^ ^]^ Thus, we further investigated the octahedral tilt/rotation of the LMO sublayers, which plays a decisive role in determining the Mn─O─Mn bond.

**Figure 6:**
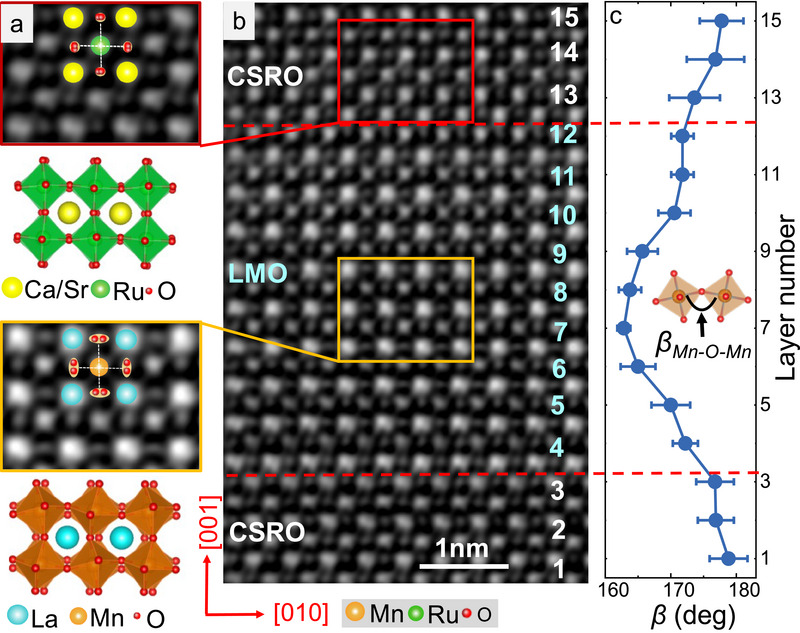
Lattice structure analysis. a) From top to bottom: an enlarged inverted ABF image of the CSRO layer, a cartoon showing the slight tilt/rotation of the RuO_6_ octahedra, an enlarged inverted ABF image from the middle range of the LMO layer, and a cartoon showing the strong tilt/rotation of the MnO_6_ octahedra. Oxygen site splitting is highlighted by the overlaid cartoons on the enlarged inverted ABF images. b) Inverted ABF image of the cross‐section of CSRO_5_/LMO_9_ SL. Dashed lines are CSRO/LMO interfaces. The scale bar in the bottom right corner represents 1 nm. C) Layer‐dependence of the tilt angle *β*_Β‐O‐B_, obtained by averaging 30 unit cells per layer along [010] direction, where B represents Ru or Mn.

**Figure** **6**a,b illustrates the inverted annular bright‐field (ABF) image, recorded by STEM along the [100] zone axis of the typical sample CSRO_5_/LMO_9_. In the idealized case without octahedral tilt/rotation, the oxygen site should be a circular spot that is located right in the middle position between two A‐site (or B‐site) cations. FromFigure 6a it can be seen that the oxygen sites in either the CSRO or the LMO layers have been deformed into ellipses. This feature is particularly prominent in the middle range of the LMO layer. It is an indication of the tilt/rotation of the RuO_6_ and MnO_6_ octahedra. In this case, one oxygen site splits into two horizontal or vertical spots as shown in the schematic diagram overlaid on the ABF image. As a consequence, the final oxygen spot looks like an elongated ellipse. To quantitatively characterize the tilt/rotation degree of the octahedra, the convolutional peak finding using a 2D Gaussian function was first adopted to determine the positions of A‐site and B‐site ions. As for the splitting oxygen sites, the positions of the two oxygen anions can be deduced from the two peaks in the line profiles that cross the oxygen ellipses (Figure S14, Supporting Information). The Ru‐O‐Ru and Mn─O─Mn bond angles, denoted by *β_Ru‐O‐Ru_* and *β_Mn─O─Mn_*, are further determined from the split oxygen sites, then the layer‐by‐layer variation of the B‐O‐B bond angle can be obtained by an average of 30 unit cells per layer as shown in Figure S14 (Supporting Information).Figure 6c shows the layer‐by‐layer variation of the *β_Ru‐O‐Ru_* and *β_Mn─O─Mn_* bond angles. As expected, the octahedral tilt/rotation is severe in the middle region of the LMO layer, resulting in a minimal *β*_Mn─O─Mn_ of ≈163°, which is similar to the bond angle of the LMO plain film.^[^ ^58^ ^]^ For the LMO monolayers in the interfacial region, however, the MnO_6_ octahedra display a mild tilt/rotation due to their coupling with RuO_6_, and a *β*_Mn─O─Mn_ of 170°–172° is obtained in the monolayers that are ≈2 u.c. away from the interface. As for the RuO_6_ octahedra, they show a relatively small tilt/rotation, and the corresponding *β*_Ru‐O‐Ru_ angle is 172°–179°. Therefore, CSRO transfers its octahedral tilt/rotation to adjacent LMO monolayers via interlayer coupling, stabilizing the high *T*_C_ state of adjacent LMO monolayers. We believe that the weakened octahedral tilt/rotation at the interface is responsible for the increase of the *T*_C_ of the LMO sublayer. However, how the tilt/rotation of the octahedra affects octahedral distortion and the exchange interaction between magnetic ions requires further investigation. Moreover, according to the PNR results, the magnetic moment of the interfacial CSRO layer is 2 *µ*_B_/Ru, indicating that the Ru ions are in the high‐spin state, different from the CSRO bare film. Similar high‐spin states have been reported for (111)‐oriented SRO films or BaTiO_3_/SrRuO_3_/BaTiO_3_ heterojunctions,^[^ ^59^, ^60^, ^61^, ^62^ ^]^ for which the strain‐caused RuO_6_ octahedron distortion or interface coupling was believed to be the reason for the appearance of high spin state Ru ions. The distortion of RuO_6_ octahedra strongly affects the Ru‐O bond length and Ru‐O‐Ru bond angle, regulating the hybridization between the 4*d* orbitals of Ru and the *p* orbitals of O anions as well as the splitting energy Δc_f_ between the *t*_2g_ and *e*_g_ orbitals. As shown inFigure 6c, the coupled tilt/rotation of oxygen octahedra at the CSRO/LMO interface has resulted in a considerable decrease of the Ru‐O‐Ru bond angle in the CSRO interfacial layer, which means a relatively large tilt/rotation thus a concomitant distortion of the RuO_6_ octahedra. In this case, the degeneracy of *t*_2g_ and *e*_g_ orbitals will be removed and the splitting energy between the *t*_2g_ and *e*_g_ orbitals will be reduced,^[^ ^63^ ^]^ stabilizing the high spin state of the CSRO interfacial layer.

Based on the above analysis, we are able to give a simple picture of the magnetic proximity effect in the CSRO/LMO SLs. An interlayer exchange interaction takes place at the CSRO/LMO interface. It could be a superexchange interaction between interfacial Ru and Mn ions as observed in the SrRuO_3_/LMO and SrRuO_3_/La_0.67_Sr_0.33_MnO_3_ heterojunctions.^[^ ^46^, ^64^, ^65^ ^]^ It makes the magnetic moment of the CSRO interface layer antiparallel to that of the LMO. As a result, each CSRO layer is divided into two interfacial layers and one inner layer, with different magnetic directions and magnetization. Interface engineering improves the FM order of the LMO layer by expanding the Mn─O─Mn bond angle, and the improved LMO layer magnetizes the CSRO layers in return.

## 3 Conclusion

In summary, SLs composed of CSRO and LMO layers have been systematically investigated based on various techniques, and a novel interfacial phase has been obtained in the CSRO layer sandwiched by LMO layers. This phase is metallic and FM over a wide temperature range and exhibits the highest Curie temperature (*T*_C_ ≈ 300 K) among the 4*d*/5*d*‐TMOs. Due to the presence of strong SOC, the $\sigma_{xy}^{AHE}$ of CSRO/LMO SLs is obviously larger than that of 3*d* oxide La_2/3_Sr_1/3_MnO_3_ films in the whole temperature range. As for the comparison with SrRuO_3_ bare films or 5*d* oxide SrIrO_3_, the $\sigma_{xy}^{AHE}$ of the interfacial CSRO phase displays an obvious advantage in the temperature range from 150 to 280 K. Particularly, this interfacial phase shows a substantially enhanced magnetization (≈2 *µ*_B_/Ru at 10 K) that is three times as large as that of the inner phase (≈0.7 *µ*_B_/Ru at 10 K), a greatly reduced coercive force (≈0.05 T at 5 K), a strong AHE below 280 K, and an in‐plane magnetic easy axis. All these features are specifically important for the room‐temperature application of this phase in oxide spintronics/orbitronics. Meanwhile, a strong improvement in FM order is also observed in the LMO layer. As revealed by the technique of XMCD, the CSRO interfacial layer and the LMO layer are antiferromagnetically coupled together, forming a ferrimagnetic arrangement of magnetic structure at the interface. As evidenced by the results of STEM, the interlayer coupling of the MnO_6_ and RuO_6_ octahedra causes an expansion of the interfacial Mn─O─Mn bond angles from ≈163° to ≈172°, facilitating the enhancement of the Curie temperature of the LMO layer. The magnetic exchange between the interfacial layers of CSRO and LMO is antiferromagnetic, as indicated by the results of PNR and XMCD, and the effects of interface coupling are transferred to the interfacial layer of CSRO, stabilizing its high *T*_C_ state. The present work demonstrates an effective approach for exploring new magnetic states in TMOs with coupled degrees of freedom, paving the way toward the design of advanced spin injection and spin‐charge interconversion devices.

## 4 Experimental Section

### Sample Synthesis

High‐quality epitaxial (CSRO_n_/LMO_9_)_8_ SLs were grown on (001)‐oriented LSAT single crystalline substrates by the pulsed laser deposition (KrF, λ = 248 nm), where *n* = 4, 5, 6, 8, and 9 represent the numbers of unit cells for the CSRO sublayer. The beginning layer is LMO and the ending layer is CSRO. CSRO and LMO bare films were also prepared for comparison. In the growth process, the substrate was kept at 650 °C, and the oxygen pressure was set to 40 Pa. The fluency of the laser pulse was 1.2 J cm^−2^ and the repetition rate was 2 Hz. After deposition, the sample was cooled down to room temperature with an oxygen pressure of 200 Pa. The deposition rate of each film was carefully calibrated by the technique of small angle X‐ray reflectivity (XRR), and the thickness of sublayers was controlled by counting the pulse number.

### Sample Characterization

The crystal structure was determined by a high‐resolution X‐ray diffractometer (D8 Discover, Bruker) with Cu‐K*α* radiation. Atomic‐scale lattice images were recorded by a high‐resolution scanning transmission electron microscope (STEM) with double C_S_ correctors (JEOL‐ARM200F). Cross‐sectional thin samples for STEM analysis were prepared using a dual‐beam focused ion beam system along the [100] direction. The magnetic properties were measured by the Quantum‐Designed vibrating sample magnetometer (VSM‐SQUID) in the temperature range of 5–300 K. The transport measurements were performed in a designed physical property measurement system (PPMS) with standard Hall bar geometry.

### Soft X‐ray Magnetic Circular Dichroism (XMCD)

Soft X‐ray absorption spectroscopy (XAS) measurements were performed in the total electron yield (TEY) detection mode at Beamline BL07U of the Shanghai Synchrotron Radiation Facility (SSRF). The XMCD spectra of the Mn *L*_2,3_ edge were defined by the difference of the XAS spectra measured with right‐handed and left‐handed circularly polarized lights, respectively. During the measurements, a magnetic field of 7 T was applied, forming an angle of 45° with film normal. The spin and orbital magnetic moments of the Mn atoms were estimated by using the XMCD sum rules.

### Polarized Neutron Reflectometry (PNR)

PNR experiments were performed at MR beamline of the Chinese Spallation Neutron Source (CSNS). The samples were field‐cooled and measured at 1.2 T along the in‐plane direction. PNR measurements were carried out at 10 K in the specular reflection geometry with wave vector transfer (*q*) perpendicular to the surface plane. The neutron reflectivity was recorded as a function of *q* for the spin‐up (*R* ^++^) and spin‐down (*R* ^−^) polarized neutrons. These neutron reflectivities were normalized to the asymptotic value of the Fresnel reflectivity (*R*_F _= 16π^2^/*q* ^4^) for a better illustration. The difference between *R* ^++^ and *R* ^−^ was calculated as the spin asymmetry SA = $\frac{\left(R^{++}-R^{--}\right)}{\left(R^{++}+R^{--}\right)}$. The PNR data were fitted using the GenX software.

## Conflict of Interest

The authors declare no conflict of interest.

## Supporting information



Supporting Information
