# Supplementary material for: Near Room‐Temperature Ferromagnetic Phase Achieved in 4d Ruthenate via Interfacial Ferrimagnetic Coupling and Octahedral Rotation Engineering
Source: Adv Sci (Weinh). 2025 Jun 19;12(33):e02613. doi: 10.1002/advs.202502613 (PMC12412501; doi:10.1002/advs.202502613)
Supplement: Supplementary file 1 — Supporting Information [file ADVS-12-e02613-s001.docx]

Supplementary Materials for

Near room-temperature ferromagnetic phase achieved in 4*d* ruthenate via interfacial ferrimagnetic coupling and octahedral rotation engineering

Mengqin Wang, Qinghua Zhang, Xiao Deng, Jie Zheng, Wenxiao Shi, Yunzhong Chen, Baogen Shen, Yuansha Chen*, Tao Zhu*, Fengxia Hu*, and Jirong Sun*

Mengqin Wang, Qinghua Zhang, Jie Zheng, Wenxiao Shi, Yunzhong Chen, Baogen Shen, Yuansha Chen, Tao Zhu, Fengxia Hu, Jirong Sun

Beijing National Laboratory for Condensed Matter Physics and Institute of Physics, Chinese Academy of Sciences, Beijing 100190, China
*Corresponding author Email: yschen@iphy.ac.cn (Y.S.C); tzhu@iphy.ac.cn (T.Z)；fxhu@iphy.ac.cn (F.X.H); jrsun@iphy.ac.cn (J.R.S)

Mengqin Wang, Qinghua Zhang, Jie Zheng, Wenxiao Shi, Yunzhong Chen, Baogen Shen, Yuansha Chen, Tao Zhu, Fengxia Hu, Jirong Sun

School of Physical Sciences, University of Chinese Academy of Sciences, Beijing 100049, China

Xiao Deng, Tao Zhu

Spallation Neutron Source Science Center, Dongguan 523803, China

Baogen Shen

Ningbo Institute of Materials Technology & Engineering, Chinese Academy of Sciences, Ningbo, Zhejiang, 315201, China

Fengxia Hu

Songshan Lake Materials Laboratory, Dongguan, Guangdong 523808, China

**Figure S1.** Electron energy loss spectroscopy (EELS) element mapping around the La-*M*_4_,_5_, Ca-*L*_2_,_3_, Mn-*L*_2_,_3_, Sr-*L*_2_,_3_, Ru-*M*_4_,_5_, and O-*K* edges for the CSRO_5_/LMO_9_ SL, recorded along [100] zone axis.

**Figure S2.** Mn *L*_2,3_ and Ru *M*_2,3_ XAS spectra of the (CSRO_2_/LMO_2_)_8_ (denoted as 2-2) and (CSRO_3_/LMO_3_)_8_ (denoted as 3-3) SLs. For comparison, the corresponding spectra of the LMO and CSRO bare films are also presented. It is found that the Mn and Ru ions of the SLs show exactly the same valence state as that of the LMO and CSRO bare films, without any signatures of interlayer charge transfer.

**Figure S3.** Saturation anomalous Hall resistivity as a function of temperature for the CSRO_n_/LMO_9_ SLs. The inset plot shows the Curie temperature of the CSRO_n_/LMO_9_ SLs, as a function of the layer thickness of CSRO. Dashed line shows the Curie temperature of the bare LMO film, ~30 u.c. in thickness.

**Figure S4.** Thermomagnetic curves of the Ca_0.5_Sr_0.5_RuO_3_ and LaMnO_3_ bare films grown on LSAT (30 u.c. in thickness), measured with a magnetic field of 0.05 T adopting the field-cooling mode.

**Figure S5.** Low-field closed view of the first ferromagnetic phase for the CSRO_8_/LMO_9_ SL.

**Figure S6.** Anomalous Hall resistivity as a function magnetic field of the CSRO bare film on LSAT (30 u.c. in thickness) measured at different temperatures. The coercive field is 1.86T at 2 K, and the Curie temperature is ~70 K.

**Figure S7.** Magnetic force microscope images of CSRO_9_/LMO_9_ SL at 2K under magnetic fields of 1T, 2T, and 3T respectively.

**Figure S8.** Magnetic hysteresis loops of the CSRO_4_/LMO_9_ SL, measured at 10 K with in-plane fields and out-of-plane fields, respectively. It is found that the sample reaches the saturation state more rapidly when the field is applied in the film plane, indicating that the magnetic easy axis lies in-plane.

**Figure S9.** AMR behavior of the CSRO bare film grown in LSAT (30 u.c. in thickness), obtained at 2 K by rotating magnetic field in the (001) plane while keeping the magnitude of the magnetic field at 3 T. Symbols: experiment results. Red line is a result of curve fitting based on the formula $AMR=a_{2}\times cos\left( 2\theta-\omega_{2} \right)+\left| a_{3}\times cos\left( 2\theta-\omega_{3} \right) \right|$. Blue and green dashed lines are the contributions of the first and second terms, respectively.

**Figure S10.** Magnetic moment as a function of magnetic field for the samples of CSRO_n_/LMO_9_ SLs with n=4, 5 and 9, measured from 10 K to 300 K. The following line corresponds close view of the *M-H* curves in low field range.

**Figure S11.** (a-c) Results of curve fitting of the *M-H* curves to the Langevin function for the CSRO_9_/LMO_9_ SL at 250 K (a), 280 K (b) and 300 K (c). (d) Magnetic moment as a function of temperature for the sample of CSRO_9_/LMO_9_, measured with an in-plane field of 0.05 T in field cooling mode.

**Figure S12.** XAS spectra around Mn *L*_2,3_ edge for the CSRO_9_/LMO_9_ SL, measured with temperature ranging from 10 K to 300 K. The red and blue lines are the spectra obtained by the circularly left-polarized light and circularly right-polarized light, respectively. The green colored lines are the corresponding XMCD signals. The sample was first cooled to a preset temperature in a magnetic field of 0.8 T, which is the highest field of our experiment facilities, and then measured after reducing the applied filed to 0.1 T.

**Figure S13.** Thermomagnetic curves of the CSRO_3_/LMO_3_ SL, measured with a magnetic field of 0.05 T adopting the field-cooling mode.

**Figure S14.** (a) A close view of the inverted ABF image showing the existence of oxygen ellipse. Along the white line a line profile analysis was performed to determine the position of oxygen anions. (b) Results of line profiles corresponding to the blue, green and read lines, showing the distinct positions of two oxygen anions. (c) A schematic diagram for measuring the B-O-B bond angle.
